# Sensitivity of reaction time to the magnitude of rewards reveals the cost-structure of time

**DOI:** 10.1038/s41598-019-56392-0

**Published:** 2019-12-27

**Authors:** Kai Steverson, Hui-Kuan Chung, Jan Zimmermann, Kenway Louie, Paul Glimcher

**Affiliations:** Center for Neural Science, New York University, 4 Washington Place, Room 809, New York, NY 10003 USA

**Keywords:** Cognitive control, Decision

## Abstract

The Drift-Diffusion Model (DDM) is the prevalent computational model of the speed-accuracy trade-off in decision making. The DDM provides an explanation of behavior by optimally balancing reaction times and error rates. However, when applied to value-based decision making, the DDM makes the stark prediction that reaction times depend only on the *relative* utility difference between the options and not on absolute utility magnitudes. This prediction runs counter to evidence that reaction times decrease with higher utility magnitude. Here, we ask if and how it could be optimal for reaction times to show this observed pattern. We study an algorithmic framework that balances the cost of delaying rewards against the utility of obtained rewards. We find that the functional form of the cost of delay plays a key role, with the empirically observed pattern becoming optimal under multiplicative discounting. We add to the empirical literature by testing whether utility magnitude affects reaction times using a novel methodology that does not rely on functional form assumptions for the subjects’ utilities. Our results advance the understanding of how and why reaction times are sensitive to the magnitude of rewards.

## Introduction

Decision making often involves not only choosing which option to select but also *when* to decide. More time spent examining the options often provides more accurate estimates of their value, which allows for better decisions. However, time spent deciding may be costly: it likely requires cognitive effort and delays receiving the benefits of the chosen option. When going to a restaurant, reading the menu more carefully may result in choosing a better meal, but it delays receiving the food and requires higher cognitive effort. Balancing the costs and benefits of when to decide is typically referred to as the “speed-accuracy trade-off”, a widely used and powerful lens through which decision making has been studied^1–3^.

One of the most widely used computational models for understanding the speed-accuracy trade-off in decision making has been the Drift-Diffusion Model (DDM), frequently employed in memory research and perceptual decision making^4,5^, and more recently in value-based decision making^6–8^. Under certain assumptions, the DDM provides a Bayesian foundation by implementing a theoretically optimal procedure that balances reaction times and error rates^9,10^. In this way, the DDM makes explicit the computational problem its algorithm optimizes, which, is a key step to understanding the mechanism as a whole^11^.

However, in the case of value-based decision making, the assumptions required for the DDM to be an optimal solution are quite restrictive. For example, they require that the utility (or value) difference between the options be fixed and known ahead of time by the decision maker. This assumption is satisfied in some perceptual tasks (e.g., the random dot motion choice experiments^12^), but not in many value-based tasks (e.g., experiments involving choosing between randomly drawn snack options^6^). Recent work by Tajima *et al*.^13^ relaxes some of these restrictions, constructing a new optimal framework for the speed-accuracy trade-off in value-based decision making that is not based on the DDM.

The model by Tajima *et al*., however, maintains one of the starkest predictions of the DDM–that reaction times only depend on the *relative* utility difference between the options presented. For example, suppose a decision maker is making a single choice between two options with utilities of 15 and 20. The DDM and Tajima’s model both predict that the decision maker would show an identical reaction time distribution as when the options have utilities 10015 and 10020. We refer to this manipulation, which increases the utility magnitude of the options while leaving the utility difference fixed, as *value scaling*. This stark prediction runs counter to some previous experimental studies that have found that value scaling decreases reaction time^14,15^. The observation that value scaling decreases reaction time is also consistent with results from perceptual decision making where the quantities being manipulated are visual features such as luminance^16,17^.

In this paper, we are interested in studying if and how it could be optimal for reaction times to decrease with value scaling, as has been observed. We present an algorithmic framework that builds on the work of Tajima *et al*., and we show that the optimality of the empirically observed pattern depends crucially on the functional form for the cost delay. These theoretical results allow us to use the study of value scaling to reveal aspects of the underlying computational problem human behavior is designed to solve. Motivated by this insight, we add to the empirical literature by testing whether value scaling impacts reaction times using a novel methodology that does not rely on functional form assumptions for the utilities of the subject.

## Theory

We show that the invariance of reaction times to value scaling in the framework of Tajima *et al*. arises from the structure of the cost of time they employ. They model the cost of delay as an *additive flow cost* this is a cost structure in which passing second imposes a fixed cost to the decision-maker and that this fixed cost is not influenced by the utilities of the options under deliberation. Under this assumption, value scaling does not interact with the cost of delay, and hence leaves the optimal reaction time unchanged as overall utility scales as long as the utility difference remains constant. As we demonstrate in the theoretical results section, this conclusion of theirs holds for a broad class of additive flow cost models.

An alternative form for the cost of delay is multiplying the utility of each option by a discount factor that decreases with time. This type of time preference we refer to simply as “discounting” and we note that it is the standard form in studies of self-control and addiction^18^, developmental psychology^19^ as well as most of economics. We call this form “multiplicative discounting” to clearly distinguish it from the additive flow cost embedded in the model of Tajima *et al*. Under multiplicative discounting, each passing second imposes a cost that is proportional to the utilities of the options. Therefore, valuing scaling increases the decision maker’s impatience and incentivizes faster decision making as overall utility increases as the utility difference is held constant. We note that the multiplicative functional form we used in this paper borrowed an idea from exponentially weighted series approaches, including the literature on temporal discounting of choices. Of course our interests here are not in temporal discounting choices nor is this a model of temporal discounting, merely borrowed functional form – albeit a form with a very different set of rates and values. Our model does not focus on understanding the decision process for temporal discounting choices such as whether people decide faster for immediate rewards or not. Instead, we proposed a model for optimal decision making that simply shapes the functional form of time discounting, albeit with a very different rate constant. Our main theoretical result demonstrates that value scaling leads to faster reaction times for a broad class of optimal multiplicative discounting models, including the exponential and hyperbolic functional forms common in the aforementioned literatures that use discounting.

**Figure 1:**
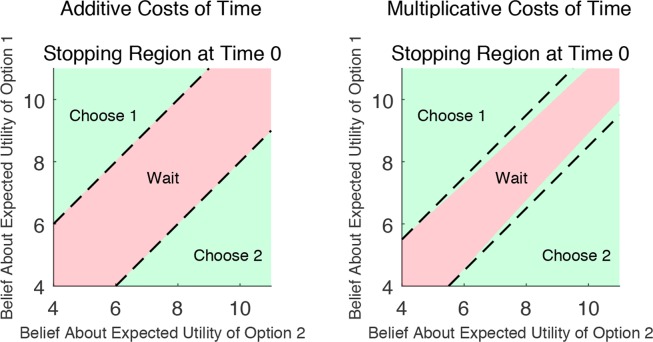
Schematic illustration of decision boundaries at time point 0 for additive and multiplicative costs of time. At each time point, the DMs beliefs about the expected utility of the two options determines they decide to wait (pink region) for more information or stop and choose (green regions). For additive cost of time (left), at each point in time, there are two boundaries parallel to the 45 degree. For multiplicative cost of time (right), what changes is that boundaries not only collapse across time, but also collapses as a funciton of value at a single time point.

Another way to understand the difference between the multiplicative and additive costs of time is through the idea of collapsing boundaries. In Fig. 1, we demonstrate this with simulation results that compare the stopping regions of the additive and multiplicative costs models, at a single point in time. At each point in time, the DMs beliefs about the expected utility of the two options determines whether they decide to wait (the pink area) for more information or stop and choose (the green area). Tajima *et al*. show that, the additive cost of time model provides a foundation for DDM-like boundaries that collapse over time. In that paper, they consider the case where there are two options available. At each point in time, the DMs beliefs about the expected utility of the two options determines whether they decide to wait for more information or stop and choose. They show that, at each point in time, there are two boundaries parallel to the 45 degree line, if the subjects beliefs lie within these boundaries then they wait and if their beliefs lie outside they choose (see Fig. 1 left). Tajima and colleagues also show that these boundaries get closer together over time, narrowing the region in which the DM waits. Hence, as argued by Tajima *et al*., these boundaries are akin to the collapsing boundaries in the DDM model and provide a optimal foundation for this feature. In our multiplicative cost of time model, what changes is that boundaries not only collapse across time, *but also collapse at a single time point* (see Fig. 1 right). Notice how the optimal waiting region (the pink area) of the right portion of Fig. 1 narrows towards the upper-right of the graph. This means the region where waiting is optimal shrinks as the expected utility of the two items increases. We can imagine starting with two options that have expected utilities ${V}_{L}$ and ${V}_{R}$, which represent one point in the graph of Fig. 1 right. We can implement value scaling by adding a fixed magnitude of $\alpha  > 0$ to both ${V}_{L}$ and ${V}_{R}$ This would move the point towards the upper-right corner in a manner parallel to the 45 degree line. Hence, as *α* increases the beliefs moves into a narrower portion of the pink region, which can result in leaving the pink region entirely. Hence, value scaling can flip the optimal decision from waiting to choosing immediately. This demonstrates how value scaling makes the DM less patient and less willing to wait. In other words, these collapsing boundaries arise due to the same logic as discussed above: raising the expected return of the two options makes the DM more impatient, even when holding the time fixed. Hence, we can think of the multiplicative cost of time as adding collapsing boundaries in a new dimension. This gives another way to view the difference between the additive and multiplicative costs, in a way that relates to the DDM.

Our theoretical results allow us to restrict the functional form for the cost of delay by studying how value scaling affects reaction time. In other words, we can reveal aspects of the computational problem human behavior is designed to solve through studying the impact of reward magnitudes on behavior. This insight motives our empirical study where we add to the literature on how reactions times vary with value scaling.

Notably, if faster decisions lead to obtaining more rewards, then higher utility magnitudes very naturally incentivize faster decision making. In this case, reaction times are no longer insensitive to value scaling in the Tajima *et al*. model or in more recent normative versions of the DDM^10^. However, in both our theoretical model and our experimental test, we focus on the case where the number of decision and prizes are fixed so that the DDM and Tajima model both predict reaction times are invariant to value scaling.

## Empirics

In the empirical portion of this paper, we add to the literature on whether reaction times actually do vary with value scaling. A direct way of implementing value scaling, used in Hunt *et al*.^14^, is to assume a parameterized functional form for the subjects’ utility function and estimate the parameters for each subject using a preliminary two-alternative choice task. The estimated parameters can be used to calibrate choice sets that all have the same utility difference between the two alternatives but different utility magnitudes, hence implementing value scaling. However, this method requires high confidence in the functional form assumption made for the utility. Any mis-specification in this functional form may lead to choice sets that do not implement value scaling by having varying utility difference across the two options. Other studies take a different tack by only studying choices where the decision maker is indifferent between the two option^15^. However, this raises the question of whether the results apply away from indifference.

In response to these concerns, we develop a novel methodology that implements value scaling without relying on indifference or functional form assumptions about utility. In our task, subjects performed a two-alternative forced-choice over lotteries. We use a manipulation of the lottery probabilities that, under the assumptions of expected utility, achieve value scaling for any possible utility function. Our methodology does assume the subjects employ expected utility theory or something equivalent to it in our carefully selected set of choices. To maximize the likelihood of this, we require all outcomes to occur with probabilities between twenty and eighty percent, a domain where expected utility theory has been shown to reasonably model observed behavior^20,21^.

Our experimental results show that value scaling significantly decreases reaction time. Our manipulation increases the expected value of each option by 3 dollars, which we estimate to have the same impact on reaction time as increasing the expected value difference between the options by 1.08 dollars. This shows that the size of the value scaling effect is on the same order as changes in the value difference. Within our algorithmic framework, our results support the conclusion that the subjects face a multiplicative discounting cost of time instead of a linear flow cost.

Our results also give support for a number of mechanistic decision making models which allow for reaction time to vary with value scaling, including certain versions of the DDM (such as those that track attentional effects)^22^; decision field theory^23^; and leaky accumulator models^24^. The fact that these models allow for value scaling effects immediately relates them to the algorithmic frameworks discussed in this paper, a point to which we return in the discussion.

## Results

### Theoretical results

We begin by presenting a framework for optimal value-based decision making that gives predictions on how reaction times should vary with value scaling. The framework involves a “cost of delay” for which we will consider two forms: additive flow cost and multiplicative discounting. We model value scaling as a constant utility increase to all possible options in all possible states. Hence, value scaling changes the *utility magnitudes* without altering the *utility differences* between the options. We show that reaction times are invariant to value scaling under additive flow costs but decrease with value scaling under multiplicative discounting.

In our framework, a decision maker (DM) chooses from a finite set of options *X*, the utilities of which are not precisely known to him. For example, the possible options might be “choose the left option” or “choose the right option”. The longer the DM takes to study his options, the more he learns about their utility. However, waiting is costly, and the DM must balance the cost of delay against the value of more information.

We impose a fixed time limit after which the DM must decide. We can set the time limit large enough to never be practically binding, e.g., 1000 years. Imposing a time limit allows us to avoid certain technical issues that complicate the analysis without adding any additional insight.

The utility of the options and the DM’s learning process are determined by a state $\omega$. The DM does not know the state directly but knows the probabilistic process governing which state is chosen. Knowledge of this probabilistic process represents the DM’s prior information. We make almost no assumptions on the shape of the DM’s prior beliefs, except that the expected value of each option is finite. For example, we can allow the values of the options to be normally distributed, which is a common assumption in these types of models. The DM’s utility from choosing option *x* in state $\omega$ is given by $U(x,\omega )+\alpha$. The parameter $\alpha$ is our value scaling term, and increasing $\alpha$ is what we mean by value scaling. The decision maker knows the shape of his own utility function, including both *U* and $\alpha$.

Over time, the DM receives signals about the utility of each option. The longer he waits, the more signals he accumulates and the more information he has to make his choice. For each option *x*, the DM observes a stochastic process ${Z}_{t}^{x}$ described by the following stochastic differential equation

1 $$d{Z}_{t}^{x}(\omega )=(U(x,\omega )+\alpha )dt+\Sigma (x)\cdot d{B}_{t}(\omega ).$$

The expected change of this process over times is $U(x,\omega )+\alpha$, which equals the utility of option *x*. Therefore, the DM can extract information about that option’s utility over time. However, the signal is muddied by noisy Gaussian errors captured by the term $\Sigma (x)\cdot d{B}_{t}$. Formally, ${B}_{t}$ is a vector of independent Brownian motions, and $\Sigma (x)$ is a vector of weights that determines how the elements of ${B}_{t}$ are weighted for option $x$.

At each point in time, the DM calculates his expected utility for each option using Bayesian updating to integrate his prior belief with the signals observed so far. Letting ${{\mathbb{Z}}}_{t}$ denote all the signals received up until time $t$, the DM’s expected utility from choosing option $x$ is given by $\alpha$ plus

2 $${m}_{t}(x,{{\mathbb{Z}}}_{t})=E[U(x,\omega )|{{\mathbb{Z}}}_{t}].$$

When the DM decides, he chooses the option with the highest expected utility according to his beliefs at that time, and that highest utility equals

3 $$\mathop{{\rm{\max }}}\limits_{x\in X}\,({m}_{t}(x,{{\mathbb{Z}}}_{t})+\alpha ).$$

Since the DM is Bayesian, the expected utility that he calculates for each option equals the expected utility he actually receives. Of course, this does not mean the DM knows the true utility exactly, only that he must be right on average.

The DM’s decision of when to decide is given by what we call a reaction time process $\tau$, which determines whether to decide at each point in time as a function of the signals received up until that point. In probability theory, this type of process is called a *stopping time*. Since we are building an algorithmic framework, we assume the DM acts according to the *optimal* reaction time process, the conditions for which we will give shortly.

We must first specify the cost of delay to the DM, for which we will consider two possibilities: additive flow cost and multiplicative discounting. Under the additive flow cost assumption, there is a strictly increasing function continuous $C:[0,\infty )\to {\mathbb{R}}$ where $C(t)$ is the cost of waiting until time $t$.

Therefore, the DM’s payoff from adopting reaction time process $\tau$ when the value scaling constant is $\alpha$ is given by

4 $${W}_{{\rm{flow}}}(\tau ,\alpha )=E[\mathop{{\rm{\max }}}\limits_{x\in X}\,({m}_{\tau }(x,{{\mathbb{Z}}}_{\tau })+\alpha )-C(\tau )].$$

The first term in the above equation is the expected utility of the selected option when the time of decision is set according to reaction time process $\tau$. The second term, $C(\tau )$, gives the cost of delay under $\tau$. Therefore, the right-hand side of the above equation equals the DM’s expected utility from the chosen option minus the cost of delay.

The optimal reaction time process maximizes ${W}_{{\rm{flow}}}(\tau ,\alpha )$ among all such processes. Since $\alpha$ is independent of the state and the chosen option, it is just a scaling constant that can be pulled out of the above equation. Therefore, value scaling does not change the optimal reaction time process, under the additive flow cost model. We formalize this insight in the following theorem.

#### **Theorem 1**.

*For any α*, *α*′, *reaction time process* $\tau$ *is optimal in the additive flow cost model with value scaling parameter α if and only if it is optimal in the additive flow cost model with value scaling parameter α*′.

Under the multiplicative discounting model, the discount factor is described by a strictly decreasing continuous function $D:[0,\infty )\to [0,\infty )$. If a choice is made at time *t*, the utility of the chosen option (including the value scaling parameter) is multiplied by $D(t)$. We also restrict the utilities of the agent to be strictly positive so that decreasing $D(\,\cdot \,)$ is a cost and not a benefit. The DM’s payoff from reaction time process $\tau$ is given by

5 $${W}_{{\rm{disc}}}(\tau ,\alpha )=E[D(\tau )(\mathop{{\rm{\max }}}\limits_{x\in X}\,{m}_{\tau }(x,{{\mathbb{Z}}}_{\tau })+\alpha )].$$

In this model, $\alpha$ is multiplied by the discounting term and therefore *does* interact with the reaction time. The longer the reaction time, the more $\alpha$ is discounted. Hence, larger $\alpha$ makes the DM more impatient and leads to faster decision making.

#### **Theorem 2**.

*Suppose α*′ > *α and suppose that*, *under the multiplicative discounting model*, $\tau$ *is optimal with value scaling parameter α*, *and* $\tau ^{\prime}$ *is optimal with value scaling parameter α*′. *Then* $\tau ^{\prime} \le \tau$ *with probability one*.

Theorems 1 and 2 provide strong predictions on how value scaling will impact reaction times under the two forms for the cost of delay. *With an additive flow cost*, *value scaling has no impact on reaction time*. *With multiplicative discounting*, *value scaling decreases reaction times*. In this way, value scaling provides insight into how to construct an algorithmic framework for the speed-accuracy trade-off. The proof of theorems is provided in the Theoretical Method.

### Experimental results

**Figure 2:**
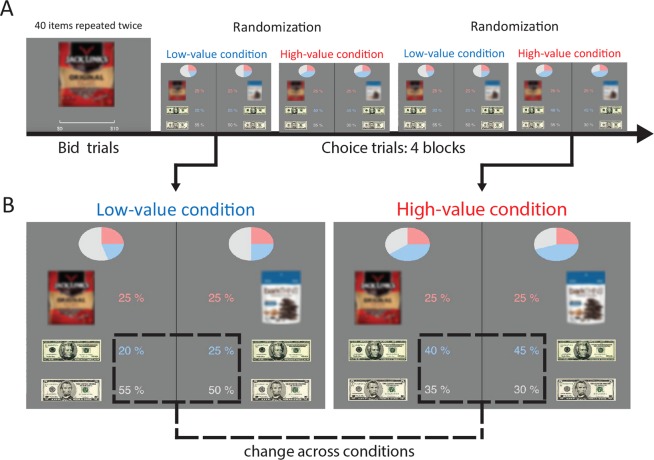
Experimental procedure. Note: The product images shown here have been blurred at the request of the journal to protect the copyright images. In the actual experiment the images were not blurred. (**A**) Time-line of the experiment. Subjects bid on 40 food items twice. The bids were used to construct 22 binary choice sets. Subjects completed four blocks where each of the 22 choice sets repeated four times within each block. One of the first two blocks and one of the second two blocks were assigned to a “low-value condition”, while the remaining blocks were assigned to a “high-value condition”. Pre-randomization was used to ensure the order of the block conditions was counterbalanced across subjects. (**B**) Screenshot of the same choice set in the low-value condition (left) and the high-value condition (right). In the high-value condition, the probability of $20 was increased by 20 percent, and the probability of $5 was decreased by 20 percent in all options.

Motivated by our theoretical results, we empirically test the impact of value scaling on reaction time in human subjects. Eighty subjects performed a two-alternative forced-choice task choosing between lotteries (Fig. 2), and reaction times were recorded on every trial. To make our procedure incentive compatible, a single trial was randomly selected at the end of the experiment, and the subject was awarded the outcome from that trial. Each lottery had three possible outcomes: a snack good, twenty dollars, or five dollars. Subjects first performed an auction task to elicit their subjective monetary values on forty snack goods to ensure that they liked the snack goods that constituted the rewards in the lotteries. For each subject, only snack goods in the top half of elicited values were used in the following lottery choice task.

In our task, the speed of decision making does not impact the reward rate since the number of trials was fixed and only a single trial was realized. This aligns with our theoretical framework, and, as discussed in the introduction, leads to the DDM and the model of Tajima *et al*. to be invariant to value scaling.

The choices over lotteries were split into four blocks. One of the first two blocks and one of the last two blocks were assigned to a “low-value” condition. The remaining two blocks were assigned to a “high-value” condition. Pre-randomization was used to counterbalance the four possible orderings of the block conditions across subjects. Identical choices were presented in each block, except in the high-value condition twenty percent probability was added to every twenty dollar outcome and subtracted from every five dollar outcome.

Our manipulation implements value scaling without assuming any particular utility function, since our high-value condition adds a fixed amount to the expected utility of each lottery, relative to the low-value condition. For example, let $$U(\$5)$$ and $$U(\$20)$$ indicate the utilities of $5 and $20 dollars for a specific subject. Then, for that subject, the change in expected utility between the conditions is $$0.2(U(\$20)-U(\$5))$$. Notably, this manipulation works without knowing the values of $$U(\$5)$$, $$U(\$20)$$, or the utility of the snack good. The only assumption we make is that more money is always better than less, which is required to know which condition is “high-value” and which is “low-value”.

Previous studies have shown that choice behavior violates expected utility theory in some contexts^25^. To address this concern, we restrict our lotteries so that each outcome occurs with probabilities between twenty and eighty percent. The probability weighting function has been shown to be nearly linear in this range implying that expected utility fits behavior under this restriction on probabilities^20,21^.

**Figure 3:**
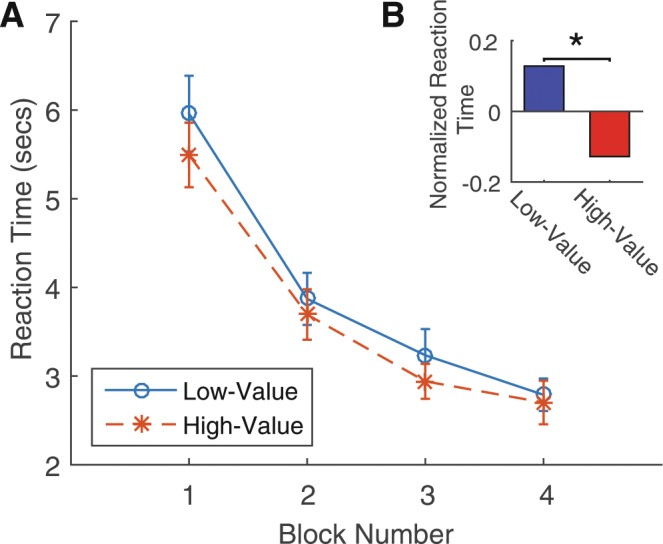
Reaction times by block order and condition. (**A**) Reaction time plotted against block order in high and low-value conditions. Error bars are calculated by clustering at the subject level. (**B**) Paired t-test shows normalized reaction times significantly lower in the high-value condition. Normalized reaction time removes the time trend by subtracting the average reaction time across all subjects from the same chronological block. Average normalized reaction time by high and low-value condition were calculated for each subject. Paired t-test was performed on these subject-level averages across the two conditions.

**Figure 4:**
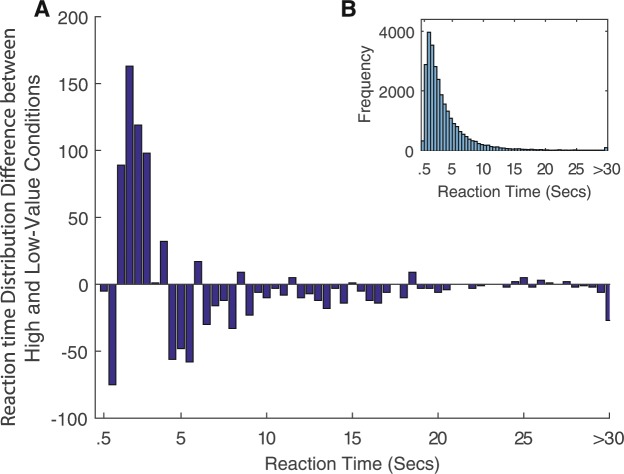
Distribution of reaction time. For both graphs, each bin represents 0.5 seconds. (**A**) Distribution of reaction time difference between the two conditions shows a higher frequency of shorter reaction time trials in the high-value condition compared to the low-value condition. Total difference across all bins sums to zero since trials are balanced across conditions. (**B**) Distribution of total reaction time across all conditions and all subjects. The mean reaction time was 3.84 s.

Our findings show reaction times decreased with value scaling consistent with our multiplicative discounting model and against the predictions of the DDM and the additive flow cost models. Mean reaction time was 3.71 s in the high-value condition and 3.96 s in the low-value condition, and 3.84 s across all trials. Figure 3 reports average reaction time across subjects for the high and low-value conditions by each block arranged in chronological order. Reaction times were lower in the high-value condition at each stage and also displayed a downward time trend. For statistical analysis, we first normalized the reaction times to remove the time trend by subtracting the population average reaction time in that chronological block. A paired t-test calculated on subject level averages showed normalized reaction times significantly lower in the high-value condition (*t*(79) = 2.53, *p* = 0.013, two-tailed, *Cohen*’*s dz* = 0.283). The difference in the reaction time distribution between our two conditions is plotted in Fig. 4.

**Figure 5:**
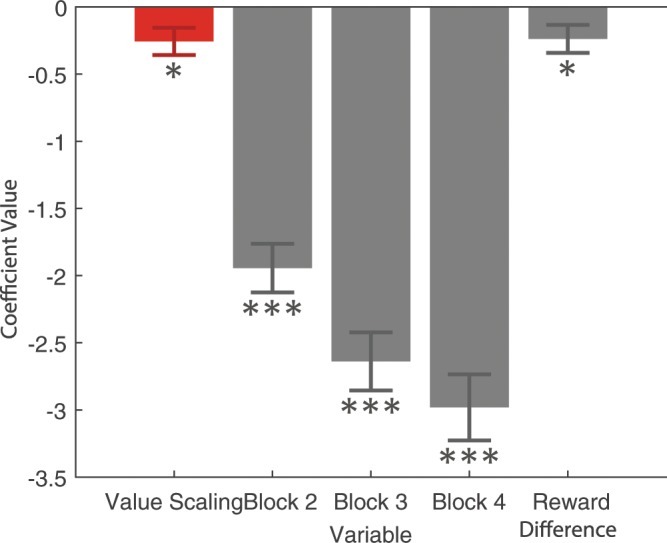
Reaction time regression. Value scaling has a negative effect on reaction time–subjects responded faster in the high-value condition. Data included all blocks and subjects. Dummy variables for each subject and a constant were included in the regression, but omitted from the figure. Standard errors were clustered at the subject level. One, two, and three stars indicates significance at 0.05, 0.01, and 0.001 levels respectively.

To further verify the result and quantify the value scaling effect, we did a trial-by-trial regression on reaction time, pooling data from across all subjects. Coefficients from the regression are reported in Fig. 5. Controls were included for the chronological number of the current block, the absolute expected reward difference between the lotteries, and each subject. The calculation for expected reward is given in the methods. Standard errors were clustered at the subject level, which corrects for correlation across trials within a subject^26^.

$$\begin{array}{rcl}RT & = & {\beta }_{0}+{\beta }_{1}{\rm{Value}}\,{\rm{Scaling}}+{\beta }_{2}{\rm{Block2}}+{\beta }_{3}{\rm{Block3}}\\  &  & +\,{\beta }_{4}{\rm{Block4}}+{\beta }_{5}{\rm{Reward}}\,{\rm{Difference}}+{\beta }_{i}{{\rm{Subject}}}_{i}\end{array}$$

In line with our paired t-test results, we find reaction times significantly decreased in the high value condition (*β* = −0.256, *p* = 0.014, two-tailed, df = 79). In other words, we find value scaling decreases reaction times, which supports the hypothesis of the multiplicative discounting model. Moreover, the size of the value scaling effect is a similar order of magnitude to changes in the value difference between the options (*β* = −0.238, *p* = 0.025, two-tailed, df = 79). Our value scaling manipulation raises the expected value of all options by 3 dollars, which are regression results estimate has the same impact of increasing the value difference between the options by 1.08 dollars.

**Figure 6:**
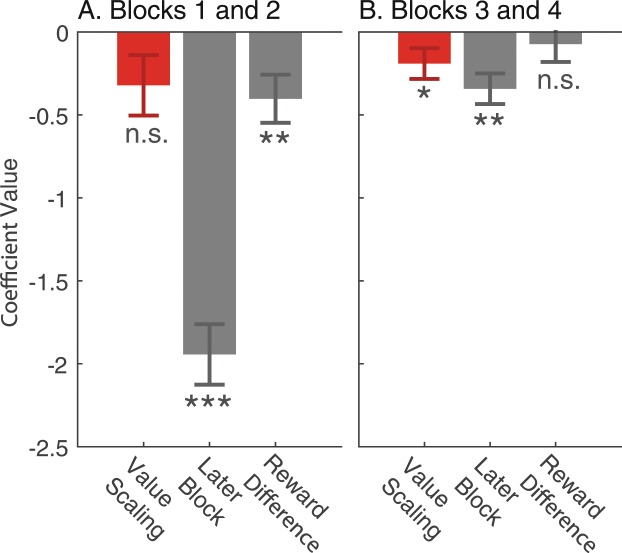
Reaction time regression, early and late blocks. Standard errors clustered at the subject level. Dummy variables for each subject as well a constant were included in the regression, but omitted from the figure. One, two, and three stars indicates significance at 0.05, 0.01, and 0.001 levels respectively. (**A**) Data from the first two blocks encountered by each subject. (**B**) Data from second two blocks encountered by each subject.

Figure 4 showed reaction time decreasing as the experiment goes on, which suggests a non-stationarity in the decision making process. Since we counterbalanced the order of high and low-value conditions, any non-stationary effect independent of value scaling would not impact our results. To examine the possibility of an interaction between the non-stationarity and our main effect, we repeated our regression on the first two and last two blocks separately. Regression coefficients are reported in Fig. 6, and all standard errors are clustered at the subject level. In both regressions, reaction times were lower in the high-value condition in the first two (*β* = −0.322, *p* = 0.08, two-tailed) and second two blocks (*β* = −0.19, *p* = 0.04, two-tailed). Since our main effect is qualitatively similar in both the later and earlier blocks, this suggest no significant interaction with any non-stationary effect.

**Figure 7:**
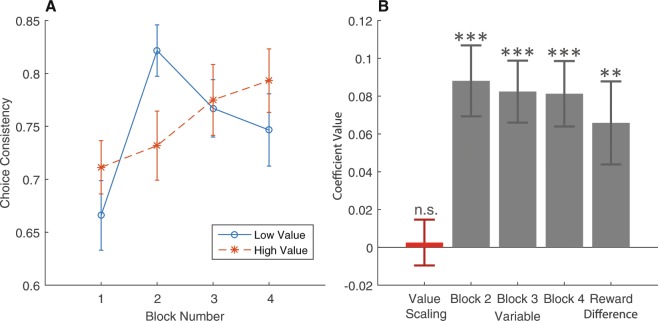
Choice consistency results. Choice consistency defined as one minus the frequency of preference reversal within a block on each choice set. Higher value indicates higher consistency in choices. (**A**) Choice consistency plotted by block order for high and low-value conditions. Data grouped across all subjects and trials. Standard errors are clustering at the subject level. (**B**) Coefficient values of choice consistency regression. Standard errors clustered at the subject level. Dummy variables for each subject were included in the regression, but omitted from the figure. One, two, and three stars indicates significance at 0.05, 0.01, and 0.001 levels respectively.

We also examined the impact of value scaling on the choice data. First, we examined the impact of value scaling on the choice consistency of the subjects. The model predicts that consistency should go down in the high-value blocks. Within each block, each pair of options is presented to the subject exactly four times. We determine whether a subject’s actions were consistent or not for each pair of options on each block. A subject’s actions are coded as consistent if she made the same decision all four times a particular pair appeared. Otherwise her actions are marked as inconsistent. Across our data set, the subjects’ actions were marked as consistent 75 percent of the time. We repeated the descriptive and regression analysis on the choice consistency variable in place of reaction time (Fig. 7). In this dataset, value scaling has no significant effect on choice consistency (*β* = 0.003, *p* = 0.833, two-tailed, df = 79), which is instead mainly driven by the block order and expected reward difference between the options (*β* = 0.066, *p* = 0.004, two-tailed, df = 79).

**Figure 8:**
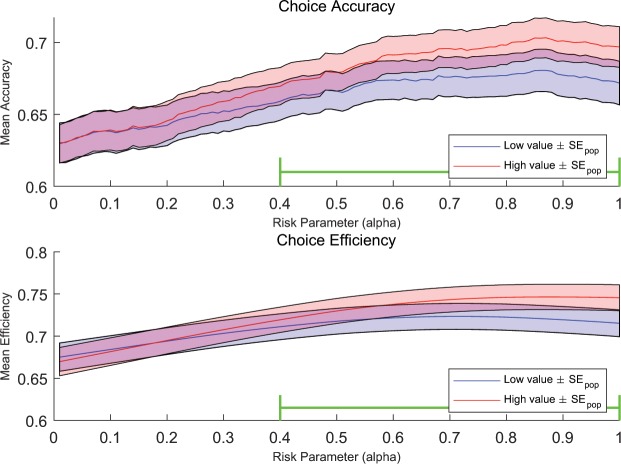
Mean choice accuracy (top) and choice accuracy (bottom) using *α* ranged 0 to 1. If the selected option with the highest expected utility, the choice was characterized as “correct” and vice versa. In this range, we see no significant difference between the choice accuracy in the high and low-value blocks. Choice efficiency measures what percent of the possible utility they achieved. In this range, we see no significant difference between the choice accuracy in the high and low-value blocks.

Second, we tested “choice accuracy”, which measures how often participants made the “correct” choice. Since our choice options are risky lotteries, which choice is “correct” depends on the participant’s level of risk aversion, which in turn depends on the shape of their utility function. Thus to perform this analysis we used one of the standard utility function forms $u(x)={x}^{\alpha }$, which has been commonly used to model decision making under risk^20,27,28^. The expected utility of each lottery for a participant is calculated as:

6 $${\rm{P}}(\$20){20}^{\alpha }+{\rm{P}}(\$5){5}^{\alpha }+{\rm{P}}({\rm{snack}}){\rm{avg}}\_{\rm{bid}}\_{{\rm{snack}}}^{\alpha },$$

where avg_bid_snack is the average of the two bids that participants made on that particular snack. Then we calculated the expected utility of each lottery using a range of possible *α*’s reported from previous studies as a fixed parameter. Thus with a given specified value for *α*, we checked to see if a given participant on a given trial selected the option with the higher expected utility, we marked that choice as “correct”, and we marked it as “incorrect” otherwise for that *α*. Choice accuracy measures the percent of choices that are “correct”. Average choice accuracy across subject for varying levels of *α* are shown in Fig. 8 top. We highlight *α*’s between 0.4 and 1, because the large majority of participants have been shown to fall in that range in previous studies^28^. In the range of *α*’s we tested, two regions are overlapped. We saw *no significant difference* between the choice accuracy in the high and low-value blocks. We note that the attempts to fit *α* at the individual subject level are not presented because the small choice set did not provide sufficient power for within subject fitting of risk attitude. When we did perform these fits they generally yielded low likelihood fits (median: 62.94%) which were accompanied by many implausible choices. In addition, only 31.25% participants have reasonable *α* estimates (convergence and in the range between 0 and 1). We believe this is due to the set of choices we used not having been designed to evenly span the choice space for stable fitting.

Finally, we also performed a similar analysis on the “choice efficiency” of the participants, which measures what percent of the possible utility they achieved. As with choice accuracy this analysis was performed across all subjects for a range of values of *α* (Fig. 8 bottom). Again, two regions are overlapped. We saw no significant difference between the high and low-value blocks, this time in choice efficiency. In brief, all three choice results show the lack of a significant effect of value scaling on choices.

## Discussion

Our paper provides three contributions. First, we reinforce the lesson from earlier work that *utility magnitude*, and not just *utility difference*, plays an important role in value-based decision making. Second, we provide an algorithmic framework that shows what type of computational assumptions would make it optimal for reaction times to be sensitive to utility magnitude. Third, we provide a methodological contribution on how to manipulate utility magnitudes without changing utility difference. Notably, our manipulation works without requiring an estimation of the utility function, and applies to cases where the subject is not indifferent between the options.

Studying the normative explanations of a behavior can reveal the computational problem that behavior is designed to solve, which can provide insight into the underlying mechanism^11,29^. In perceptual decision-making tasks such as random-dot motion (RDM) task, the optimal decision rule is specified clearly: maximize a desired level of accuracy (because only correct response will be rewarded) within minimum time spending. Thus this is a classic speed-accuracy trade-off scenario and it has been shown that the classic DDM (with fixed non-collapsing boundaries) implements optimal procedure such that the evidences are accumulated at each time frame until one threshold of the option is reached^9,10^. In this way, studying algorithmic model of value-based decision making under the framework of DDM is an important step to understand the decision process as a whole. However, it is critical for relaxing some assumptions for studying such model. In perceptual decision-making tasks, the evidence (drift) has a known fixed magnitude in each trial (e.g. the coherence is unchanged within a trial and this is known to decision makers). However, in value-based decision-making, the magnitude of the drift is assumed to be the value (utility) difference between the options. Relaxing this assumption is one of the core motivations of the work of Tajima *et al*.. They showed that collapsing boundaries DDM is a result of an optimal model with additive cost of time. In this vein, our present work seeks to further understand the cost of delay in different functional forms within an algorithmic framework for the speed-accuracy trade-off in value-based decision making. We find evidence for a multiplicative discounting form that aligns with how delays are modeled in others areas of research, such as self-control and addiction. This alignment indicates a similar mechanism may be used across these areas, and suggests that techniques and ideas from those areas may be usefully imported to studying the speed-accuracy trade-off, which opens interesting possibilities for future research.

While the value scaling effect we found in our paper has been shown in perceptual decision making task and may be possibly explained by other models^16,17^, our model provides a prediction for studying value-based decision making process, which complements mechanistic explanations. Our experimental findings support mechanistic models that allow reaction times to vary with value magnitude (e.g., attentional DDM^22^, decision field theory^23^, and leaky accumulator models^24^), and argues against mechanistic models that do not (e.g., most variants of the DDM including the classical DDM^4^ and all those summarized in^6^). Our theoretical results suggest that multiplicative discounting is a way forward to build an algorithmic framework in which the mechanistic processes in the first group of models would be optimal. A worthwhile future direction is to study the psychological/neural mechanism of multiplicative discounting in speed-accuracy trade-off behavior for further normative foundation.

The main motivation for our experimental methodology was to implement value scaling without making functional form assumptions on the utility function. We note that reaction time alone is enough to fulfill our goal of distinguishing between different structures of time cost, thus we mainly focused on the impact of value scaling on reaction times but not choice data. However, we acknowledge that along with previous theoretical models, our algorithmic model also predicts that choice consistency should go down as reaction times decrease (in the high-value blocks). However, in our choice data, value-scaling had no significant effects which is somewhat puzzling. This may be because in our paradigm, each choice repeats only four times within each block, which only allows for a coarse measure of choice stochasticity which masks a small effect. Indeed, one might conclude that our method is not ideal for detecting changes in choice stochasticity. This may be why we did not observe significant results in that domain. We note that we also did not find any significant effects on choice accuracy or choice efficiency. For all these reasons, we believe investigating the impact of value scaling on choice data requires a modification of our current methodology and is left as an important direction for future work.

A feature of value scaling we have implicitly used, but not explicitly discussed, is that it impacts the entire distribution of options, and not just the options presented. The distinction here is between encountering options with higher or lower utility in a fixed environment versus changing the environment to raise the utility of all options. Our notion of value scaling is the latter one, which we implemented theoretically by shifting the utility function independent of the state, and we implemented experimentally through our high and low-value conditions. In many situations a person knows from context when the average value of the options in their environment has changed. For example, moving from a restaurant that serves a type of food the observer likes (high-value condition) to a restaurant that serves a type of food the observer likes less (low-value condition). We explicitly assume that when we signal a block shift and change the values of the offers subjects are aware of this change and use it to condition their behavior.

Finally, we note that participants were told explicitly that only one trial would be rewarded and they faced a fixed number of trials. Facing this constraint, subjects should maximize the reward from every single trial instead of going faster to earn more from completing more trials. In this regard our experiment is unlike classic perceptual decision-making tasks, where earnings are determined by the accumulated number of correct trials. In addition, subjects were required to wait with their rewards for 30 minutes after the experiment completed before leaving the laboratory. Going faster could reduce the aggregate time in the lab by several seconds, but that is all.

## Materials and Methods

### Theoretical methods

Let $(\Omega , {\mathcal F} ,{\mathscr{P}})$ be the complete probability space governing the state, where $\Omega$ is the set of states, ${\mathcal F}$ is a sigma algebra on the states and ${\mathscr{P}}$ is a probability measure. Note that the probability space must be non-atomic since Brownian motion is defined on it. Let ${\{{ {\mathcal F} }_{t}\}}_{t\in [0,T]}$ be the natural filtration (with the usual augmentation to include all null sets) adapted to the signals ${Z}_{t}^{x}$, where $T$ is the time limit by which the DM must decide. A stopping time is any function $\tau :\Omega \to [0,T]$ such that $\{\omega \in \Omega :\tau (\omega )\le t\}\in { {\mathcal F} }_{t}$ for all $t\in [0,T]$. For any option $x$ and state $\omega$ we define

7 $${m}_{t}(x,\omega )=E[U(x,\omega )|{ {\mathcal F} }_{t}(\omega )].$$

Note that if ${{\mathbb{Z}}}_{t}(\omega )$ is the set of signals received up until time $t$ in state $\omega$, then

8 $${m}_{t}(x,\omega )={m}_{t}(x,{{\mathbb{Z}}}_{t}(\omega )),$$

which shows the equivalence between our two definitions for ${m}_{t}$.

To prove Theorem 1, use the fact that the value scaling parameter (*α*) does not depend on the state to get

9 $${W}_{{\rm{flow}}}(\tau ,\alpha )=E[\mathop{{\rm{\max }}}\limits_{x\in X}\,{m}_{\tau (\omega )}(x,\omega )-C(\tau )]+\alpha .$$

From the above equation, it is immediate that the optimality of a stopping time is independent of *α* in the flow cost model, which proves Theorem 1.

To prove Theorem 2, first note that for any stopping time $\tau$ and any *α*, *α*′ we have that

10 $${W}_{{\rm{disc}}}(\tau ,\alpha ^{\prime} )={W}_{{\rm{disc}}}(\tau ,\alpha )+E[D(\tau )](\alpha ^{\prime} -\alpha ).$$

Now suppose that $\tau$ and $\tau ^{\prime}$ are optimal in the discounting model with value scaling parameters *α* and *α*′, respectively. Further suppose that $\alpha ^{\prime}  > \alpha$. Define stopping times $\underline{\tau }\,:\,=\,{\rm{\min }}\,\{\tau ,\tau ^{\prime} \}$ and $\overline{\tau }\,:\,=\,{\rm{\max }}\,\{\tau ,\tau ^{\prime} \}$. It is easy to check that $\underline{\tau }$ and $\overline{\tau }$ are valid stopping times.

Let $E=\{\omega \in \Omega :\tau (\omega ) < \tau ^{\prime} (\omega )\}$. Note that $\tau ^{\prime}$ and $\underline{\tau }$ differ only on the set *E*. $\tau$ and $\overline{\tau }$ also only differ on *E*. Moreover, on *E*, we have $\tau ^{\prime}$ equals $\overline{\tau }$, and $\underline{\tau }$ equals $\tau$. Hence, we can conclude that

$${W}_{{\rm{disc}}}(\tau ^{\prime} ,\alpha ^{\prime} )-{W}_{{\rm{disc}}}(\underline{\tau },\alpha ^{\prime} )={W}_{{\rm{disc}}}(\overline{\tau },\alpha ^{\prime} )-{W}_{{\rm{disc}}}(\tau ,\alpha ^{\prime} ).$$

Applying Eq. (10), we can transform the above display equation into

$${W}_{{\rm{disc}}}(\tau ^{\prime} ,\alpha ^{\prime} )-{W}_{{\rm{disc}}}(\underline{\tau },\alpha ^{\prime} )={W}_{{\rm{disc}}}(\overline{\tau },\alpha )-{W}_{{\rm{disc}}}(\tau ,\alpha )+(\alpha ^{\prime} -\alpha )E[D(\overline{\tau })-D(\tau )].$$

And we can rearrange the above equation into the following.

$$(\alpha ^{\prime} -\alpha )E[D(\overline{\tau })-D(\tau )]={W}_{{\rm{disc}}}(\tau ^{\prime} ,\alpha ^{\prime} )-{W}_{{\rm{disc}}}(\underline{\tau },\alpha ^{\prime} )+{W}_{{\rm{disc}}}(\tau ,\alpha )-{W}_{{\rm{disc}}}(\overline{\tau },\alpha \mathrm{)}.$$

By the optimality of $\tau ^{\prime}$ on $\alpha ^{\prime}$ and $\tau$ on $\alpha$, the right-hand side of the above equation must be weakly positive. Hence, $E[D(\overline{\tau })-D(\tau )]$ must also be weakly positive. Recall that $D(\,\cdot \,)$ is strictly decreasing and, by definition, $\overline{\tau }\ge \tau$ with probability one. Therefore, it must be that $\overline{\tau }$ and $\tau$ are equal with probability one, which is equivalent to our desired result of $\tau \ge \tau ^{\prime}$ with probability one, which proves Theorem 2.

The preceding discussion glossed over the questions of whether the expectations in the definitions of ${W}_{{\rm{flow}}}$ and ${W}_{{\rm{disc}}}$ are finite and whether an optimal stopping time exists. We now show both of these facts. For any stopping time $\tau$

$$\begin{array}{lll}{W}_{{\rm{flow}}}(\tau ,\alpha ) &  <  & E[\mathop{{\rm{\max }}}\limits_{x\in X}\,{m}_{\tau (\omega )}(x,\omega )-C(0)+\alpha ]\\  &  <  & E[\mathop{{\rm{\max }}}\limits_{x\in X}\,U(x,\omega )-C(0)+\alpha ]\\  &  <  & \infty .\end{array}$$

The last inequality follows from the fact that there are a finite number of options and $E[U(x,\omega )] < \infty$ for each $x$. Analogous arguments prove the expectations are finite in the expression for ${W}_{{\rm{disc}}}$.

We also wish to show that an optimal stopping time exists. Again, we will consider only the flow cost model, the arguments for the discounting model are entirely analogous. Note that standard regularity properties of Brownian motion are enough to ensure that ${ {\mathcal F} }_{t}$ (with the standard augmentation) is a continuous filtration. Endow the space of all functions $\Omega \to [0,T]$ with the uniform metric. We want to show that the set of stopping times is compact within this space. Boundedness is immediate due to the time limit. Now suppose that ${\tau }_{n}$ is a sequence of stopping times with limit $\tau$. Fix any time $t$. For each $\varepsilon  > 0$ and each *n* define

$$E(\varepsilon ,n)=\{\omega \in \Omega |{\tau }_{n}(\omega )\le t+\varepsilon \}.$$

Note that $E(\varepsilon ,n)\in { {\mathcal F} }_{t+\varepsilon }$, since ${\tau }_{n}$ is a stopping time. Using that ${\tau }_{n}\to \tau$ we know that

$$\mathop{\mathrm{lim}}\limits_{\varepsilon \to 0}\,\mathop{\mathrm{lim}}\limits_{n\to \infty }\,E(\varepsilon ,n)=\{\omega \in \Omega |\tau (\omega )\le t\}.$$

And since ${ {\mathcal F} }_{t}$ is right-continuous, it must follow that $\{\omega \in \Omega |\tau (\omega )\le t\}\in { {\mathcal F} }_{t}$, which shows the set of stopping times is compact.

It now suffices to show that ${W}_{{\rm{flow}}}$ is continuous in the stopping time, and then the existence of an optimal stopping time follows from the extreme value theorem. Using the continuity of the filtration with fact that the probability space is non-atomic is enough to get that, if ${\tau }_{n}\to \tau$, then with probability one

11 $$\mathop{{\rm{\max }}}\limits_{x\in X}\,{m}_{{\tau }_{n}(\omega )}(x,\omega )\to \mathop{{\rm{\max }}}\limits_{x\in X}\,{m}_{\tau }(\omega ).(x,\omega )$$

And for any collection of stopping times ${\mathscr{T}}$, the set ${\{{{\rm{\max }}}_{x\in X}{m}_{\tau (\omega )}(x,\omega )-C(\tau (\omega ))\}}_{\tau \in {\mathscr{T}}}$ is uniformly integrable, the proof of which uses a similar sequence of steps used to prove the finiteness of the expectations in ${W}_{{\rm{flow}}}$. By the properties of uniformly integrability, Eq. (11), and the continuity of *C*, we then have that

$${W}_{{\rm{flow}}}({\tau }_{n},\alpha )\to {W}_{{\rm{flow}}}(\tau ,\alpha ),$$

whenever ${\tau }_{n}\to \tau$, which proves the continuity of ${W}_{{\rm{flow}}}$, as desired.

### Experimental methods

#### Participants

Eighty-two healthy individuals participated in the experiment. Two individuals were excluded before performing the analysis due to lack of engagement with the study. The remaining eighty individuals (19–55 years old, average age: 24, 37 males) were included for the analysis. The University Committee on Activities Involving Human Subjects at New York University approved this study, and the experiment was performed in accordance with their guidelines and regulations. All participants provided written informed consent before participating.

#### Experimental task

The experimental session began with bid trials. In each bid trial, a high-resolution image of a food item was displayed on the screen and participants reported a bid for that item using a mouse-controlled slider bar; possible bids ranged from $0 to $10 in $0.01 increments. Forty different food items (common salty and sweet snack foods) were presented randomly, and each individual good was presented twice. Each subject was initially endowed with $10 for use in the bid trials.

Using the bid values of the food items, the experimental program constructed 22 choice sets for use in a two-alternative forced-choice task. Each choice set consisted of a unique pair of lotteries with up to three possible outcomes: $20, $5 and a food item. The choice sets were chosen so that every outcome had a probability that lay between 0.2 and 0.6, in order to avoid the nonlinearity, usually observed below 0.2 and above 0.8, in the probability weighting function^20,21^. The two bids on each food item were combined to create an average bid, and only food items in the top half of average bids were used in the lottery choice task. Moreover, we only used lotteries in the same choice set if the absolute difference in the expected reward difference was less than a dollar. Our calculation for expected reward of a lottery was

12 $${\rm{B}}({\rm{FI}}){{\rm{P}}}_{{\rm{FI}}}+5{{\rm{P}}}_{\$5}+20{{\rm{P}}}_{\$20},$$

where ${{\rm{P}}}_{{\rm{FI}}}$, ${{\rm{P}}}_{\$5}$, and ${{\rm{P}}}_{\$20}$ denote the probability of the lottery awarding a food item, 5 dollars and 20 dollars, respectively, and B(FI) denotes that subject’s average bid on that food item.

Using a minimum of three outcome lotteries was necessary to implement our value scaling manipulation without making any assumption about an individual’s utility function/risk attitude. Our manipulation requires that every lottery contains $5 and $20 as a possible outcome. If those were the only two outcomes, then every choice would have a dominant option: the one with a higher probability of $20. Our hypothesis thus requires a third outcome, and we elected a non-monetary third outcome to prevent participants from easily calculating expected values when making their choices. To mitigate the effect of this necessarily unusual choice set participants completed extensive practice before the main data collection task began, and verified verbally that they understood the structure of the task and the option before data collection began.

Each subject performed four blocks and each choice set repeated 4 times within each block. There were two low-value blocks and two high-value blocks. The block orders were counterbalanced between subjects. In high-value blocks, we increased the probability of $20 by 20% and decreased the probability of $5 by 20%, relative to the same choice in the low-value block. Subjects performed 352 choice trials in total. In each trial, subjects viewed two lotteries and indicated their choice by pressing either the left or right-arrow keyboard button. The location of each lottery on the screen (left or right) was randomly assigned in each trial. The task was programmed using PsychoPy (http://www.psychopy.org).

#### General procedure

Participants were told that one trial, either a bid or lottery choice, would be randomly selected from all of the trials for realization at the end of the experiment. If a bid trial was selected, the outcome was determined via a Becker–DeGroot–Marschak (BDM) auction^30^; the BDM procedure is widely used in laboratory economic studies, and the optimal strategy for subjects is to report the true price they would be willing to pay for eating that item at the end of the study. To realize a bid trial, subjects drew a chip from a bag containing chips numbered from $0$10 in $0.01 increments. If participant’s bid was higher than the drawn price, the participant purchased the good at that drawn price. If the bid was smaller than the drawn price, the participant paid nothing and did not receive the item. Participants were carefully informed of all the properties of the BDM auction in the initial instructions. If a choice trial was selected, the experimental program ran the selected lottery and the subject received the outcome.

Participants were instructed to fast for 4 hours before the experiment and informed that they would have to stay in the experimental room for 30 minutes after completing the experimental session, during which the only food they could consume was any food item received from the experiment. On a hunger scale of 1 (not at all hungry) to 4 (very hungry), participants reported average hunger of 2.91 with a standard deviation of 0.73. Participants read the instructions and were given an opportunity to ask questions and completed several practice trials to get familiar with the task. Participants performed the task by themselves, one participant per each experimental session. Most participants completed the experiment within 1.5 hours and were paid a $15 participation fee plus task earnings. All participants also filled out a simple demographic form after the experimental session.

#### Descriptive analysis of reaction time

Across all subjects, we averaged the reaction time data by block order in the low and high-value conditions separately. For reaction time distributions, we binned the reaction times into 0.5 second intervals up until 30 seconds. All reaction times above 30 seconds were combined into a single bin.

#### Reaction time regression

Across all subjects and blocks, reaction time data was regressed on value scaling, block order, absolute difference in expected reward between the two options, and dummies for each subject. An intercept term was also included. Value scaling is a dummy variable which takes value 1 in the high-value condition and 0 otherwise. The block order dummy variables indicate which block number the trial is in, with block 1 being the omitted dummy. Absolute reward difference used the definition of expected reward given in Eq. 12. Subject dummies controlled for individual differences. Standard errors were clustered at subject level. We also performed the same analysis using only the last two blocks.

#### Choice consistency

Choice consistency defined as a probability of no preference reversal within a block on each choice set. We analyzed the choice consistency data in the same way we did for the reaction time data.

### Simulation methods

To demonstrate the difference between the additive and multiplicative costs of time, we simulated both models in Fig. 1 for the optimal decision boundaries at time 0. For the additive cost model (Fig. 1 left), we used the simulation methods laid out in Tajma *et al*.^13^ see the methods section from that paper for more details). Under additive cost as used in Tajima *et al*., the value function at time t is adjusted to

13 $$V-t\ast c$$

where *c* is a fixed parameter and $t\,\ast \,c$ captures the cost of time.

For the multiplicative cost model (Fig. 1 right), the simulation methods were altered to make the cost of time multiplicative. To do so, the only change necessary is in how the value function V was adjusted by the current time step. Under a multiplicative cost of time, the value function was instead

14 $$\exp (\,-\,rt)V,$$

where *r* is a fixed parameter.

The value of *c* and *r* were set to 0.1 and 0.04 respectively. The two simulations shared all other parameters, which were as follows: Value space grid from to 0 to 15 with step size of 0.01. Time from 0 to 5 with step size of 0.05. Initial prior on the left and right option have variance 1. Variance of the noise of the diffusion process is 0.5.

## Data Availability

Data is available from the authors upon request.
